# Topical phospho-sulindac (OXT-328) is effective in the treatment of non-melanoma skin cancer

**DOI:** 10.3892/ijo.2012.1577

**Published:** 2012-07-27

**Authors:** KA WING CHENG, GEORGE MATTHEOLABAKIS, CHI C. WONG, NENGTAI OUYANG, LIQUN HUANG, PANAYIOTIS P. CONSTANTINIDES, BASIL RIGAS

**Affiliations:** 1 Division of Cancer Prevention, Department of Medicine, Stony Brook University;; 2 Medicon Pharmaceuticals Inc., Stony Brook, NY, USA

**Keywords:** skin cancer, phospho-sulindac, skin cancer treatment, hydrogel

## Abstract

Phospho-sulindac (P-S, OXT-328), a novel sulindac derivative, has shown superior anticancer efficacy and safety compared to sulindac. In this study, we investigated the efficacy of topical P-S hydrogel in the treatment of non-melanoma skin cancer in preclinical models. P-S is a potent inhibitor of A431 epidermoid carcinoma *in vitro* and achieves this effect by inhibiting cell proliferation and inducing apoptosis. The anticancer efficacy of topical and oral P-S was further evaluated in mice bearing A431 intradermal xenografts. Compared to the controls, topical P-S hydrogel inhibited the A431 xenografts by 70.5% (p<0.01), while oral P-S inhibited it by 43.4% (p<0.05), being significantly less effective than topical P-S (p= 0.017). Topical P-S hydrogel generated significant levels (>500 nmol/g tumor tissue) of intact P-S in the tumors, accounting for 92.5% of the total metabolites in the A431 xenografts. This local delivery of high levels of intact P-S to the A431 xenografts is an important contributor to the potent activity of topical P-S and no local or systemic side effects were noted in the treatment group. Thus, topical P-S is a promising treatment modality against non-melanoma skin cancer and merits further evaluation.

## Introduction

Non-melanoma skin cancer is the most common type of cancer in Caucasian populations (1,2). In the United States alone, two million people are diagnosed with non-melanoma skin cancer every year, with squamous cell carcinoma (SCC) and basal cell carcinoma (BCC) accounting for the majority of cases (3). Surgical removal is the standard therapy for the treatment of SCC and BCC, but it may cause morbidity in high risk individuals and have negative cosmetic outcomes. Thus, the development of alternative modalities for the treatment of non-melanoma skin cancer remains highly desirable.

Non-steroidal anti-inflammatory drugs (NSAIDs) have demonstrated significant efficacy in the chemoprevention of colon (4) and skin cancer (5,6). However, their use is limited by gastrointestinal toxicity that does not justify prolonged use in healthy individuals (7). Prompted by these concerns, our group has synthesized novel phospho-derivatives of conventional NSAIDs (8–18). Phospho-NSAIDs show superior anticancer efficacy and reduced gastrointestinal toxicity compared to conventional NSAIDs in preclinical models. P-S (OXT-328), a phospho-derivative of sulindac, is a potent inhibitor of colon cancer. The present study examined the anticancer activity of P-S towards skin cancer.

Topical treatment regimens are considered to be effective alternatives for non-melanoma skin cancer. Skin delivery is non-invasive; achieves high local levels of drug; minimizes systemic exposure; and is more acceptable to patients. In our previous investigations, the effective delivery of P-S to tumors was limited by its inactivation by carboxylesterases (9,12). We reasoned that the local delivery of P-S by topical application will bypass the liver and the intestinal tract, major sites of carboxylesterase expression, and hence, will be more effective than delivery via the oral route.

In this report, we demonstrate that P-S is a potent inhibitor of non-melanoma skin cancer cells, and topical delivery of P-S resulted in strong inhibition of skin cancer xenografts in mice. These findings suggest that topical P-S is a promising strategy for the treatment of non-melanoma skin cancer.

## Materials and methods

### Reagents

P-S (OXT-328) was a gift from Medicon Pharmaceuticals Inc. (Setauket, NY, USA). Cell culture reagents were purchased from Cellgro (Herndon, VA, USA). Other reagents, unless otherwise stated, were obtained from Sigma-Aldrich (St. Louis, MO, USA).

### Cell culture

The human epidermoid carcinoma cell line (A431) was obtained from American Type Culture Collection (ATCC), and maintained in DMEM media containing 10% fetal bovine serum and penicillin/streptomycin. All experiments were performed with cells between passages 1 to 10.

### Topical hydrogel preparation

A mixture of Pluronic P123 and P-S, dissolved in tetrahydrofuran (1:10, w/w), was dialyzed for 24 h at room temperature through a membrane (molecular weight cutoff of 3,500 Da) in phosphate-buffered saline, which was replaced three times. The dialysis bag was then wiped with absorbent paper and placed under solid PEG (MW 900,000) to absorb and concentrate the solution inside the bag until gel formation.

The final drug loading onto the gel was 1.4% (w/w), while the polymer constituted 27–30% w/v of the gel. Control gel was prepared by a cold method where 28% w/v of pluronic P123 was dispersed slowly in PBS at 2–5°C.

### In vitro cytokinetic analyses

Cell viability was measured with the MTT assay (Roche Diagnostics) and cell proliferation with the 5-bromo-2′-deoxyuridine (BrdU; BD Immunocytometry Systems) assay, according to the manufacturer’s instructions. Apoptosis and necrosis were assessed by staining cells with Annexin V and propidium iodide (PI) and analyzing them by flow cytometry (19).

### A431 xenografts

Female NOD/SCID mice (6–7 weeks-old) were purchased from Harlan Sprague-Dawley (Indianapolis, IN, USA). At 7–8 weeks of age, the mice were inoculated intradermally on both flanks with A431 cells (2x10^6^ each) suspended in 100 μl complete DMEM medium. When the average tumor size reached 120±40 mm^3^, the animals were divided into four groups (n=6–7), and were given the following treatments, respectively: i) none; ii) topical plain hydrogel (3 times per day); iii) P-S (150 mg/kg/d, p.o.); and iv) topical P-S hydrogel (50 mg/kg/d, 3 times per day). The tumors were measured twice a week with a digital microcaliper, and tumor volumes were calculated using the following formula: tumor volume = [length × width × (length + width/2) × 0.56]. After treatment for 2 weeks, the animals were sacrificed and their tumors were removed. The levels of P-S and its metabolites in the tumors were determined by HPLC. This animal study was approved by the Institutional Animal Care and Use Committee of Stony Brook University.

### HPLC analysis

The HPLC system consisted of a Waters Alliance 2695 Separations Module equipped with a Waters 2998 photo-diode array detector (328 nm) (Waters, Milford, MA, USA) and a Thermo BDS Hypersil C18 column (150×4.6 mm, particle size 3 μm) (Thermo Fisher Scientific, Waltham, MA, USA). The mobile phase consisted of a gradient between buffer A [H_2_O, acetonitrile, formic acid, 95:4.9:0.1 (v/v/v)] and 100% acetonitrile.

### Immunohistochemistry

Cell death and proliferation of paraffin-embedded A431 xenograft tissue sections were determined using the terminal deoxynucleotidyl transferase dUTP nick end labeling (TUNEL) and Ki-67 immunohistochemical staining, respectively, as previously described (19).

### Statistical analyses

Data are expressed as the mean ± SEM. Statistical analyses were performed by ANOVA. P-values <0.05 were considered statistically significant.

## Results

### Topical P-S inhibits the growth of A431 xenografts

**Figure 1:**
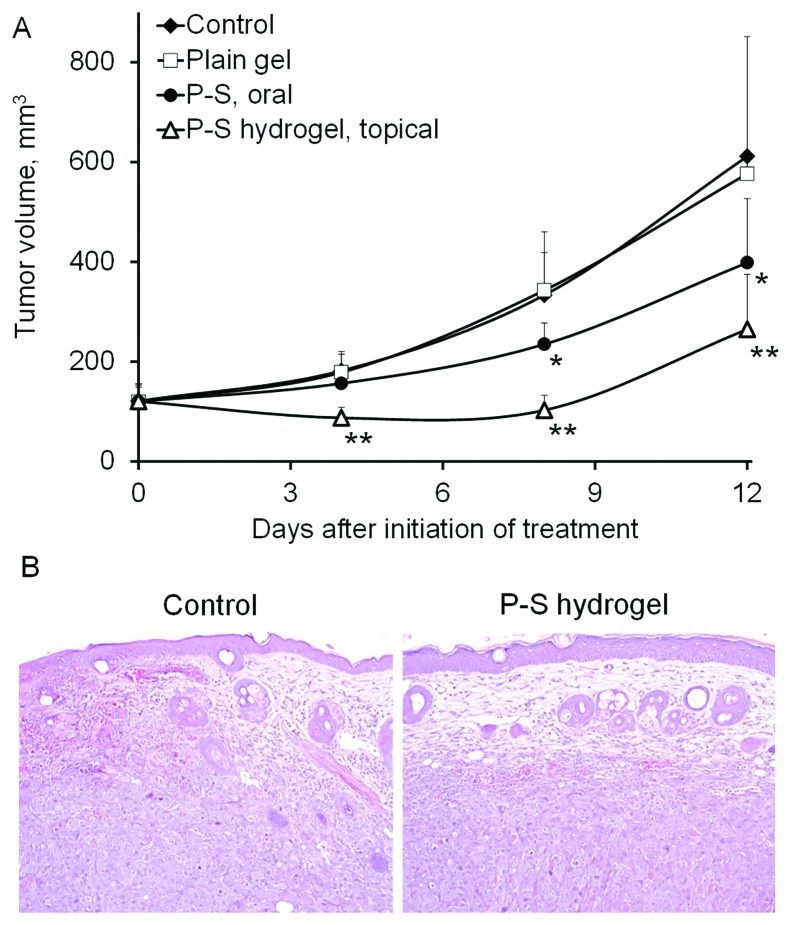
P-S hydrogel inhibits non-melanoma skin cancer growth in a xenograft model. A431 human skin cancer cells (2×10^6^) were implanted intradermally into both flanks of SCID mice. When the tumor volume reached 120 mm^3^, the mice were treated with oral P-S (150 mg/kg per day) or topical P-S (50 mg/kg, 3 times a day) for 12 days. (A) A431 tumor growth over time for the untreated control (♦), plain hydrogel (□), oral P-S (•) and topical P-S hydrogel (▵). (B) Representative images of the A431 epidermoid carcinoma (H&E): the tumor from control group shows invasive growth, while the tumor treated with P-S hydrogel is encapsulated underneath the skin. ^*^, p<0.05; ^**^, p<0.01, compared to control group.

To assess the *in vivo* efficacy of P-S against skin cancer, we treated SCID mice bearing A431 xenografts (n=6–7) topically with vehicle hydrogel, or P-S hydrogel, or orally with P-S, whereas the last group was left untreated. At equivalent dosage, topical P-S more effectively inhibited the growth of A431 tumors (p=0.017) compared to oral P-S (Fig. 1). Topical P-S caused regression of tumors during the first week. At the end of the experiment, topical P-S reduced tumor growth by 70.5% (p<0.001) relative to the control. Oral P-S (150 mg/kg/day), on the other hand, did not cause any tumor regression and modestly inhibited tumor growth by 43.4% (p<0.01). No difference in the final tumor volume was found between the vehicle and the untreated group. These findings indicate that topical P-S causes a profound inhibitory effect on the growth of human skin cancer xenografts. Body weights showed no significant difference between our two study groups (data not shown) and no local or systemic side effects were noted in the treatment group.

### P-S decreases proliferation and induces apoptosis in vitro and in vivo

To evaluate the effect of P-S on cell growth, we determined the 24 h-IC_50_ values of P-S and sulindac in A431 cells (Table I). P-S demonstrated a dramatically enhanced cytotoxicity (36-fold) compared to sulindac. In addition, it was also much more potent than the sulindac metabolites, sulindac sulfide (8-fold) and sulindac sulfone (31-fold).

**Figure 2:**
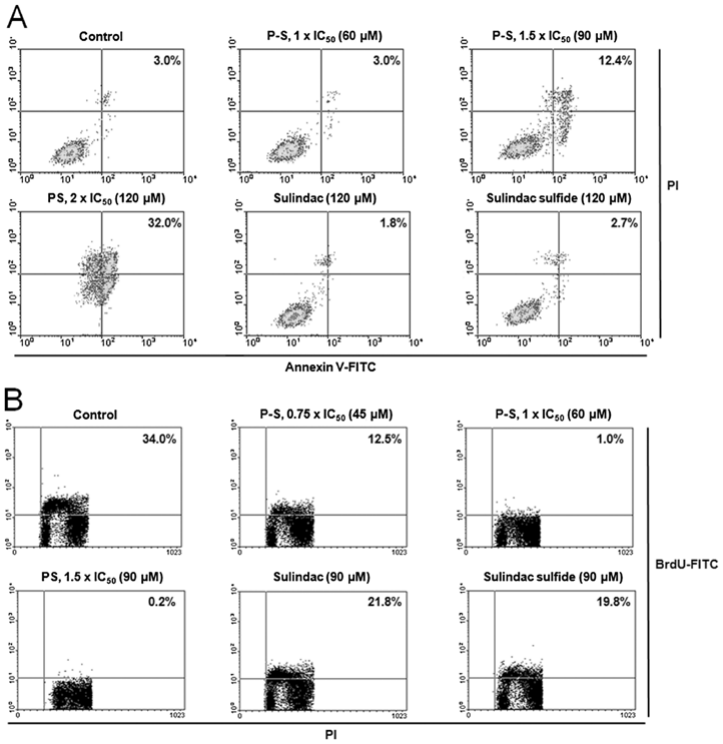
The cytokinetic effect of P-S in A431 cells. (A) A431 cells treated with P-S for 24 h were stained with PI and Annexin V and analyzed by flow cytometry. The numbers in the upper right box represent the percentage of apoptotic cells. (B) BrdU incorporation assay in A431 cells following P-S treatment for 24 h. The number in the right upper box denotes the percentage of cells in S phase.

The potent growth inhibitory effect of P-S results from its cytokinetic effect (Fig. 2). P-S concentration-dependently decreased A431 cell proliferation, reaching 97% and a near complete (>99%) inhibition at 1×IC_50_ and 1.5×IC_50_, respectively. Equimolar concentration (1.5×IC_50_ PS) of sulindac or sulindac sulfide only weakly inhibited cell growth (<40%). Cell cycle analysis revealed that PS treatment induced cell cycle arrest at G_2_/M phase (Table II).

Moreover, treatment of A431 cells with P-S at 1.5×IC_50_ and 2×IC_50_ for 24 h induced significant apoptosis to 4.1- and 10.7-fold higher than that of the control, respectively. Equimolar concentration (2×IC_50_ PS) of sulindac or sulindac sulfide had no measurable impact on cell proliferation or apoptosis.

**Figure 3:**
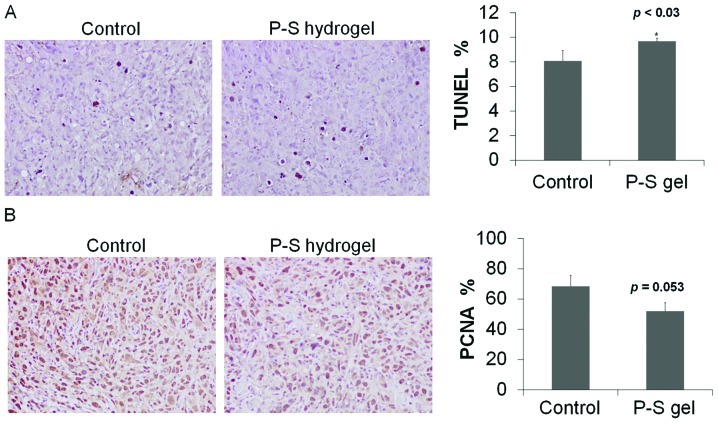
P-S hydrogel suppresses cell proliferation and induces cell death by apoptosis in human A431 xenografts in mice. (A) Images of tissue sections (upper) stained by the TUNEL method. Apoptotic (TUNEL-positive) cells were counted and expressed as the percentage of the total number of cells per field. (B) Images of tissue sections stained for PCNA expression. Proliferating (PCNA-positive) cells were counted and expressed as the percentage of total number of cells per field. ^*^, p<0.05 compared to control.

We next determined cell proliferation and apoptosis in tumor tissue sections. Compared to control, topical P-S decreased cell proliferation (determined by PNCA staining) by 25% and induced apoptosis (TUNEL) by 30% (p<0.05) (Fig. 3). These findings indicate that P-S profoundly suppresses proliferation and induces apoptosis in A431 cells, which rationalizes its potent growth inhibitory effect.

### P-S hydrogel delivers substantial amount of intact drug in vivo

**Figure 4:**
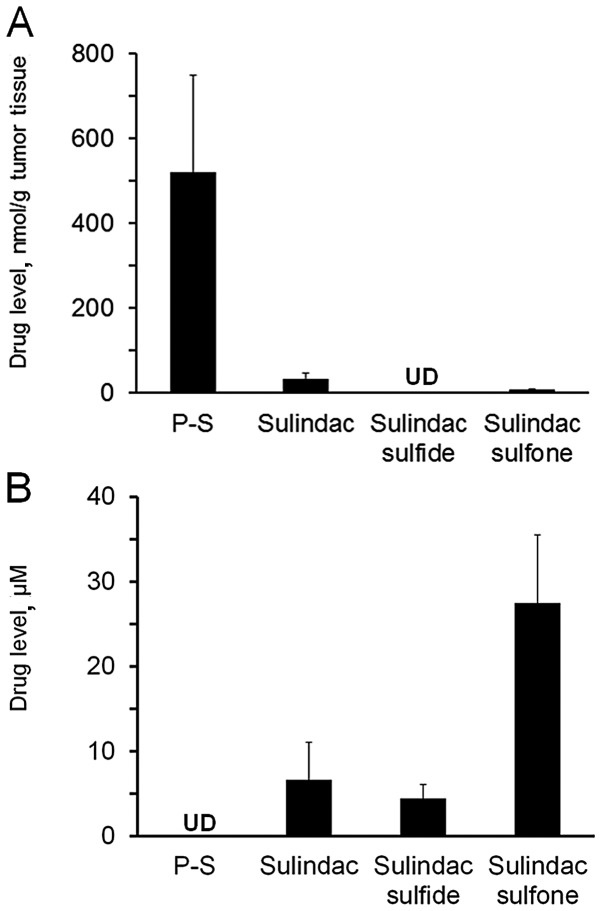
Drug levels in mice following P-S hydrogel treatment. Mice bearing A431 xenografts were treated topically with P-S hydrogel 50 mg/kg and the levels of P-S and its metabolites 1 h post-treatment were determined in (A) A431 xenografts and (B) whole blood (n=6). UD, undetectable.

To evaluate the delivery of P-S via the topical route, we measured blood and tumor drug levels one hour after drug administration (Fig. 4). Topical P-S generated significant levels (>500 nmol/g tumor tissue) of intact P-S in the tumors, accounting for 92.5% of the total metabolites in the A431 xenografts. Sulindac, sulindac sulfide and sulindac sulfone were also detected in tumors, but at much lower levels.

No intact P-S could be detected in the blood of the topical P-S treated animals. Sulindac is the major metabolite found in the blood, albeit at lower levels (7-fold lower) compared to those after oral administration (12). These data indicate that topical delivery of P-S results in high levels of intact P-S in skin tumors, which may contribute to its higher antitumor efficacy compared to oral delivery.

## Discussion

Our study demonstrates that P-S is a strong inhibitor of nonmelanoma skin cancer in pre-clinical models. Topical P-S strongly suppresses the growth of A431 skin cancer xenografts in mice, an effect mediated by i) the potent cytokinetic effect of P-S on A431 skin cancer cells, and ii) direct delivery to the skin tumors of intact P-S, the biologically most active molecule.

P-S is a potent inhibitor of the A431 epidermal skin cancer cell line *in vitro* (36-fold more potent than sulindac). A strong cytokinetic effect underpins the inhibitory potency of P-S, which is a result of inhibition of cell proliferation, induction of apoptosis and cell cycle arrest (G_2_/M). The induction of apoptosis appears to be the predominant mechanism, as P-S profoundly triggers apoptosis in A431 cells (4 to 10-fold over control) *in vitro*; whereas an equimolar level of sulindac, the parent NSAID, has no significant effect.

P-S can be incorporated in a pluronic polymer to form a hydrogel for topical application. *In vivo*, topical application of P-S strongly suppresses the growth of A431 xenografts by 70.5%, compared to the control. Oral administration of P-S, on the other hand, only resulted in a moderate inhibition of xenograft growth (43.4%). Interestingly, the anti-tumor efficacy of the topical route is significantly better than that of oral administration (differs by nearly two fold). Topical P-S also effectively induces apoptosis in A431 xenografts. These results indicate that P-S is an efficacious agent against non-melanoma skin cancer, and topical delivery of P-S appears to confer a significant therapeutic advantage compared to oral administration. Dilution effects, intestinal absorption and the metabolism of oral P-S can account for its lower efficacy.

An important contributing factor to the potent activity of topical P-S is the improved delivery of intact drug to tumors *in vivo*. P-S is considerably (8- to 36-fold) more cytotoxic towards A431 cells than its metabolites, sulindac, sulindac sulfide and sulindac sulfone (Table I), or the diethylphosphate linker (15). However, our previous investigations have shown that P-S, when given orally, was rapidly metabolized, which primarily gave rise to the above three metabolites in plasma (9,12) with minimal distribution of P-S to tumors.

Carboxylesterase 1, a broadly-specific carboxylesterase highly expressed in the intestine, liver and plasma, is primarily responsible for the hydrolytic inactivation of P-S (9,12). The presence of carboxylesterases in these organs compromises drug efficacy by converting P-S into its significantly less active metabolites. Rodent skin, on the other hand, has esterase activity over 10-fold lower than that of the liver and plasma (20). Consistent with the low carboxylesterase activity in the skin, we demonstrated that the topical application of P-S resulted in very high local levels of intact P-S (>90%) in A431 xenografts. Correspondingly, topical P-S exerts a considerably more potent growth inhibitory effect compared to P-S given orally.

Topical drug delivery is a valuable strategy of limiting systemic exposure, thereby lowering the risk of undesirable side effects. Indeed, topical therapy has been proposed to reduce the potential gastrointestinal and cardiovascular side effects of conventional NSAIDs in the treatment of actinic keratosis (21), pain (22) and arthritis (23). The topical administration of P-S to mice bearing human skin cancer xenografts resulted in 4- to 5-fold lower levels of sulindac and its metabolites (<35 μM) compared to those after oral administration (>200 μM) (12).

In conclusion, topical application of P-S-incorporating pluronic hydrogel is effective in inhibiting the growth of nonmelanoma skin cancer, and has superior efficacy compared to oral administration. Our results indicate that direct skin delivery of P-S is a promising modality for the treatment of skin cancer which merits further investigation.

## Figures and Tables

**Table I.** Inhibitory effect of P-S and its metabolites on A431 cell growth.

Compound	24-h IC_50_, μM mean ± SD	Enhancement (fold)
P-S	60.4±0.2	-
Sulindac	2,210±90	36
Sulindac sulfide	461±62	8
Sulindac sulfone	1,860±60	31

**Table II.** The effect of P-S on the cell cycle distribution of A431 cells.

	G_0_/G_1_	S %total, mean ± SD	G_2_/M
Control	43±3	26±4	30±2
P-S 60 μM	46±3	19±2	34±5
P-S 120 μM	28±2	29±6	42±7
Sulindac 120 μM	47±2	26±3	27±4
Sulindac sulfide 120 μM	49±5	24±3	26±2
